# Supplementary material for: Towards a global understanding of the drivers of marine and terrestrial biodiversity
Source: PLoS One. 2020 Feb 5;15(2):e0228065. doi: 10.1371/journal.pone.0228065 (PMC7001915; doi:10.1371/journal.pone.0228065)
Supplement: S1 Table — (DOCX) [file pone.0228065.s001.docx]

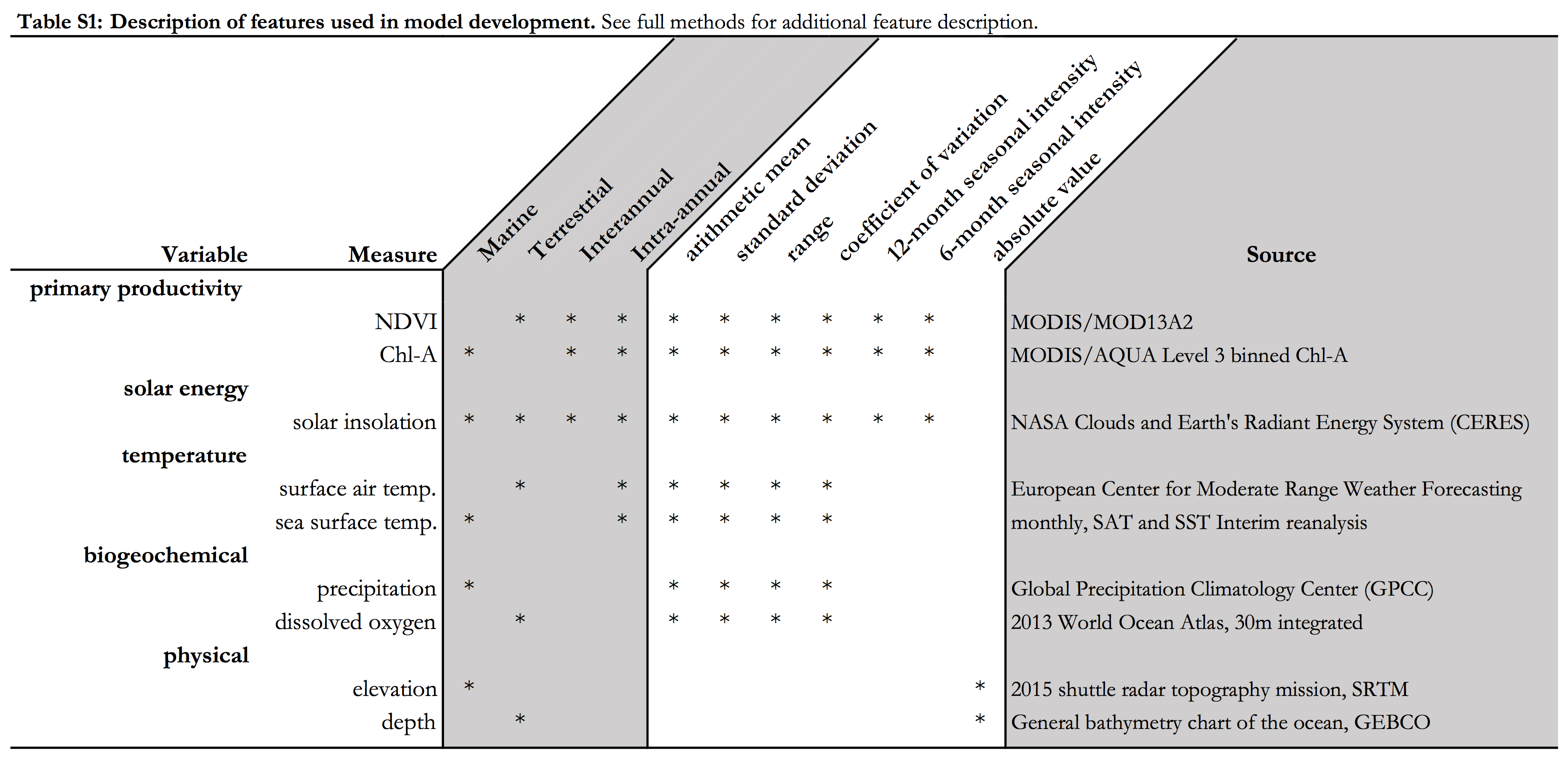


Table S1. All remotely-sensed variables are modeled from fullest temporal extent of available data streams, described in the main text Methods.
