# Supplementary material for: Towards a global understanding of the drivers of marine and terrestrial biodiversity
Source: PLoS One. 2020 Feb 5;15(2):e0228065. doi: 10.1371/journal.pone.0228065 (PMC7001915; doi:10.1371/journal.pone.0228065)
Supplement: S1 Fig — All inputs are projected in a cylindrical equal-area projection centered on 195-degree latitude. Equatorial resolution is approximately 50 km × 50 km. Gray masks are where comparable metrics were used in separate domains, i.e. SST/SAT or O2/H2O. All PP metrics are prefixed with ann for interannual metrics and sub for intra-annual metrics, AMP prefixes refer to PP seasonal wavelet intensity followed by the 6 or 12-month period. Twel_inten and six_inten refers to the seasonal wavelet intensity of solar insolation. All biogeochemical constraints (O2 and H2O) are intra-annual metrics. Variability and tendency are measured as labeled in each panel: mean, range, sd, CV. See S1 Table for summary of metrics utilized in model training. (DOCX) [file pone.0228065.s002.docx]

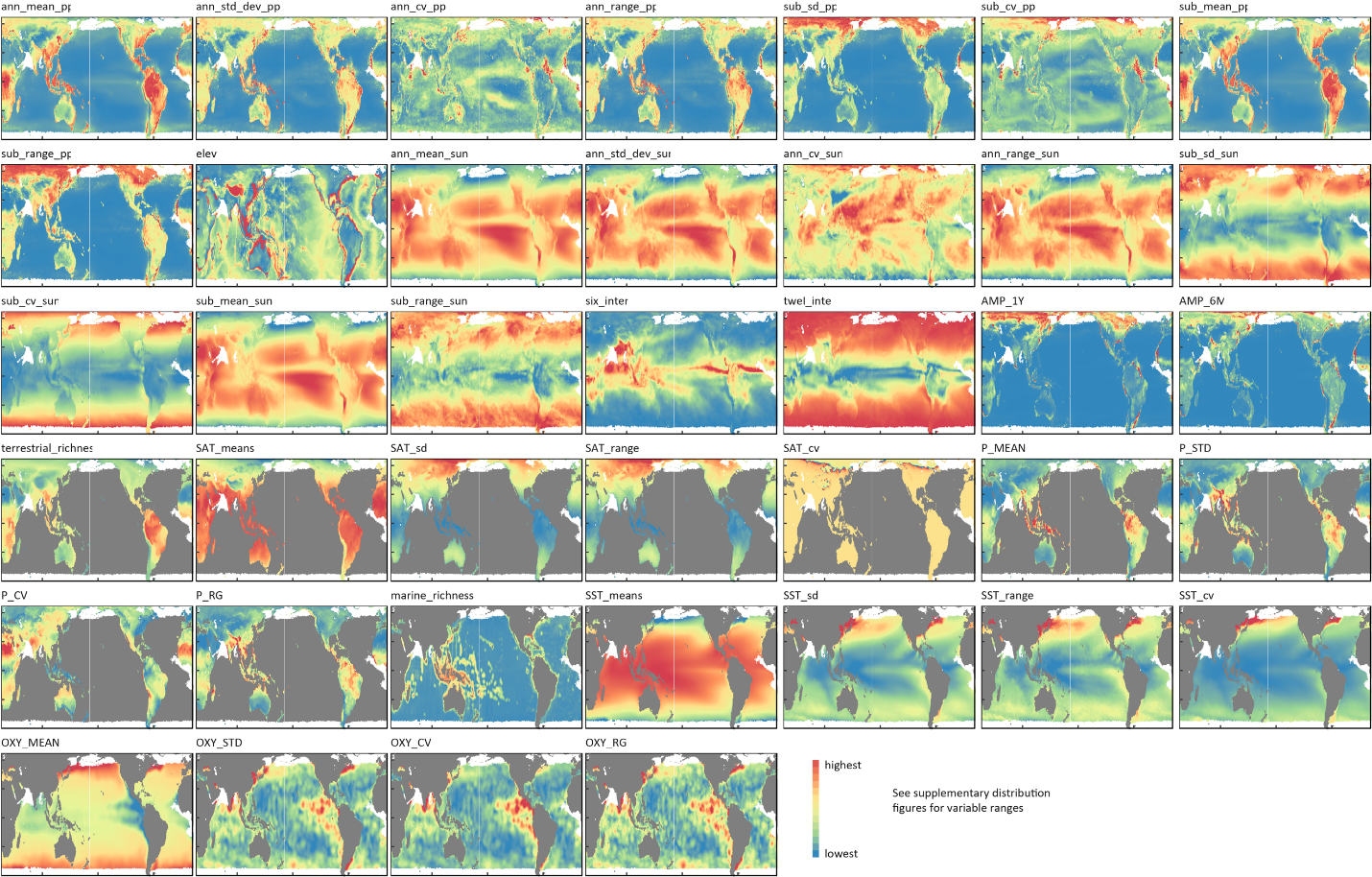


**Figure S1.** **Geographical distribution of model predictors and response.** All inputs are projected in a cylindrical equal-area projection centered on 195-degree latitude. Equatorial resolution is approximately 50 km × 50 km. Gray masks are where comparable metrics were used in separate domains, i.e. SST/SAT or O_2_/H_2_O. All PP metrics are prefixed with *ann* for interannual metrics and *sub* for intra-annual metrics, *AMP* prefixes refer to PP seasonal wavelet intensity followed by the 6 or 12-month period. *Twel_inten* and *six_*inten refers to the seasonal wavelet intensity of solar insolation*.* All biogeochemical constraints (O_2_ and H_2_O) are intra-annual metrics. Variability and tendency are measured as labeled in each panel: *mean, range, sd, CV*. See Table S1 for summary of metrics utilized in model training.
