# Supplementary material for: Towards a global understanding of the drivers of marine and terrestrial biodiversity
Source: PLoS One. 2020 Feb 5;15(2):e0228065. doi: 10.1371/journal.pone.0228065 (PMC7001915; doi:10.1371/journal.pone.0228065)
Supplement: S3 Fig — See full methods for description of development. (DOCX) [file pone.0228065.s004.docx]

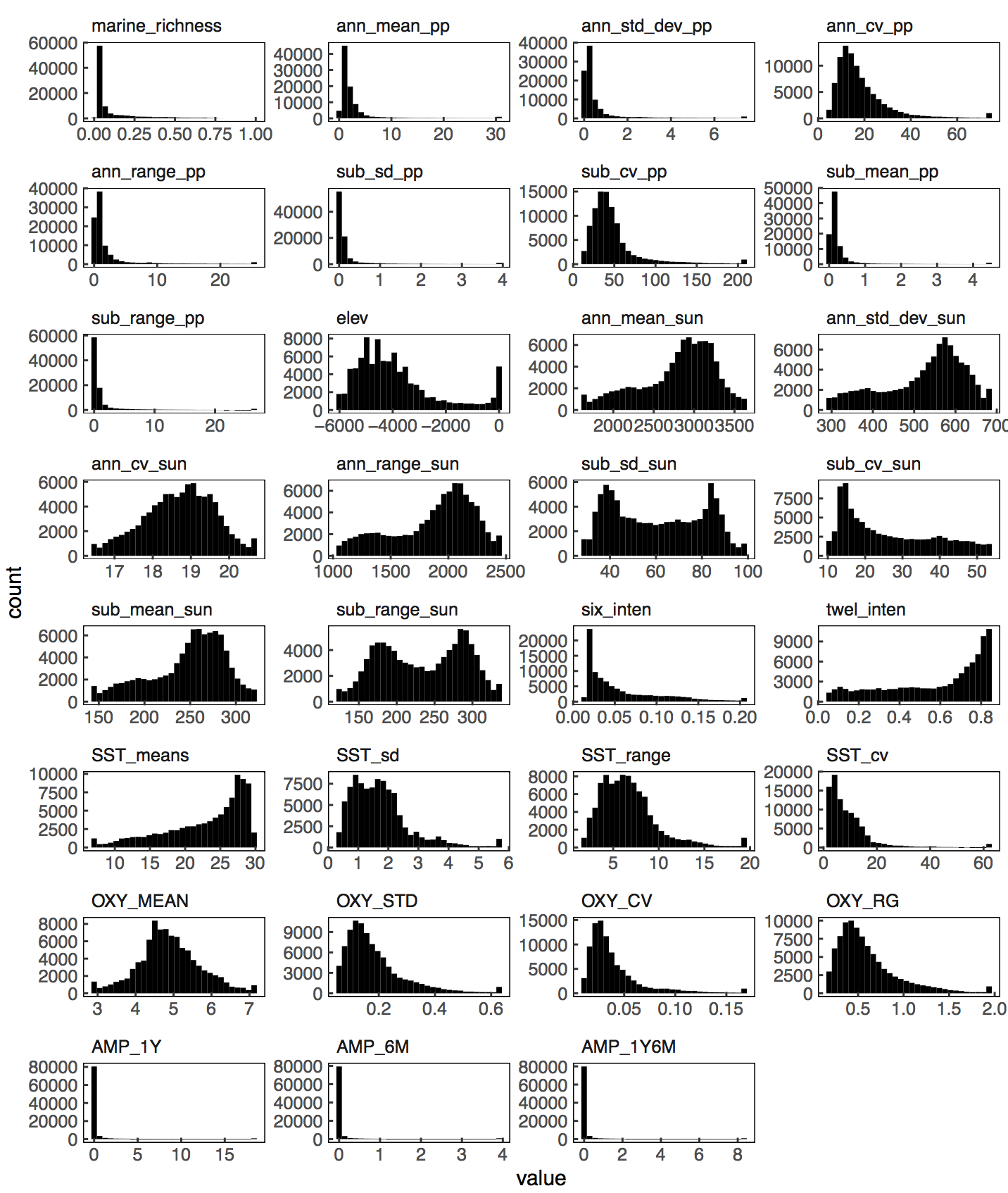


**Figure S3.** **Distributions of model features and response for the marine ANN biodiversity model.** See full methods for description of development.
