# Supplementary material for: Towards a global understanding of the drivers of marine and terrestrial biodiversity
Source: PLoS One. 2020 Feb 5;15(2):e0228065. doi: 10.1371/journal.pone.0228065 (PMC7001915; doi:10.1371/journal.pone.0228065)
Supplement: S4 Fig — Blue lines refer to marine domain and green terrestrial. The x-axis is the labeled 0–1 scaled predictor, y-axis is always scaled ln(x+1) transformed 0–1 scaled species richness. Also see S9 Fig for raster visualization of all the features shown in this figure. See caption of S1 Fig for variable naming conventions. (DOCX) [file pone.0228065.s005.docx]

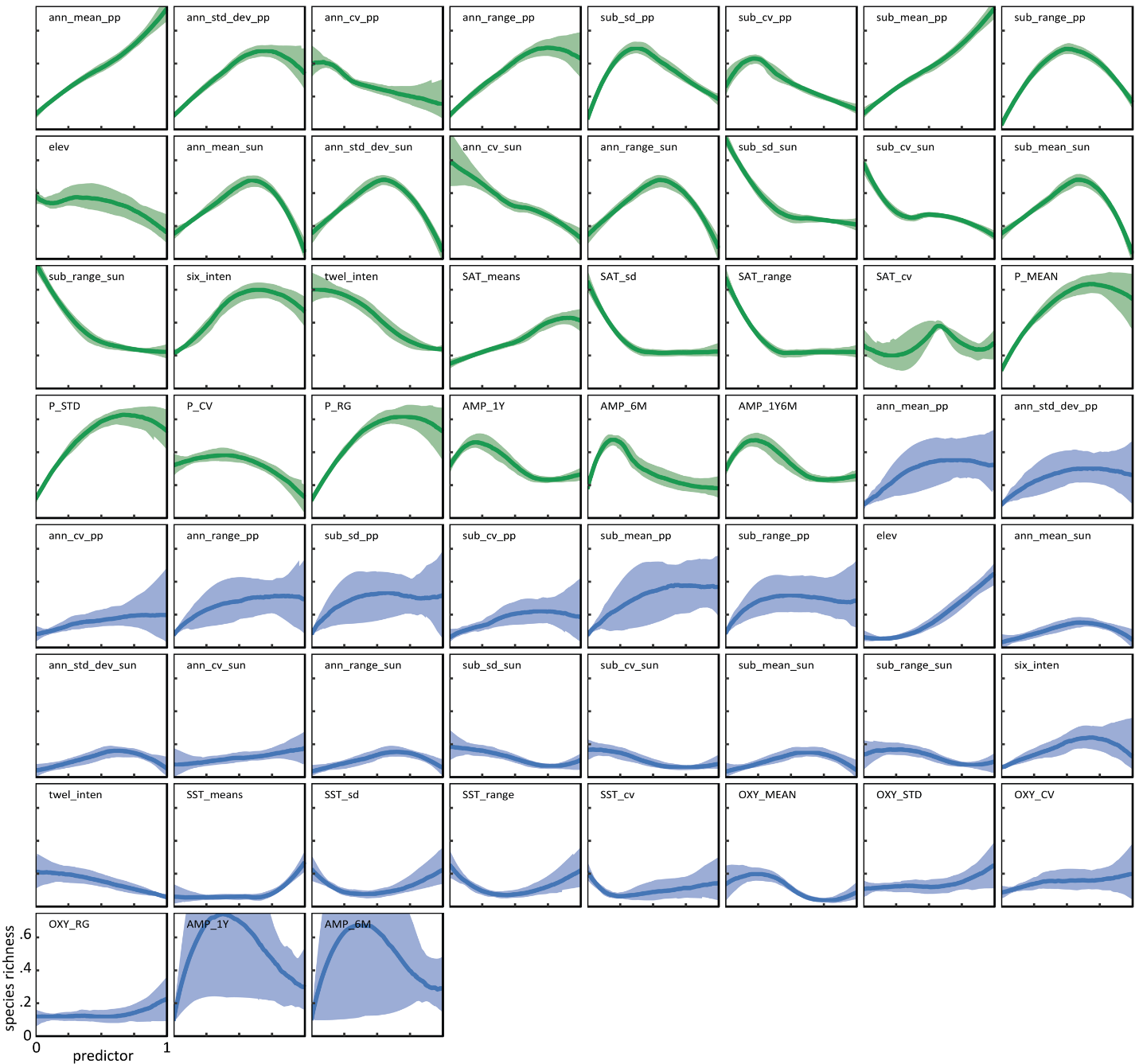


**Figure S4.** **Full compilation of model input pairwise relationships with species richness.** Blue lines refer to marine domain and green terrestrial. The x-axis is the labeled 0-1 scaled predictor, y-axis is always scaled ln(x+1) transformed 0-1 scaled species richness. Also see Figure S9 for raster visualization of all the features shown in this figure. See caption of Figure S1 for variable naming conventions.
