# Supplementary material for: Towards a global understanding of the drivers of marine and terrestrial biodiversity
Source: PLoS One. 2020 Feb 5;15(2):e0228065. doi: 10.1371/journal.pone.0228065 (PMC7001915; doi:10.1371/journal.pone.0228065)
Supplement: S5 Fig — Three examples of differing seasonal NDVI periodicities and the associated power spectrum. The continuous wavelet transformation applied to raster time series of Chl-A and NDVI is described in the methods and extensively in the annotated reference material. (DOCX) [file pone.0228065.s006.docx]

**
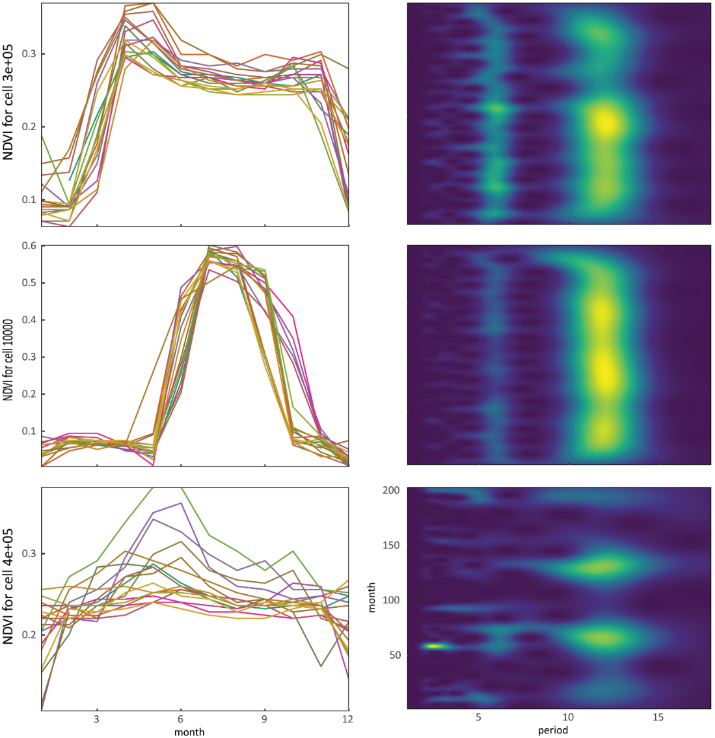
**

**Figure S5. Example approximations of 6- and 12-month phenological wavelet power with Morlet wavelet power spectrum.** Three examples of differing seasonal NDVI periodicities and the associated power spectrum. The continuous wavelet transformation applied to raster time series of Chl-A and NDVI is described in the methods and extensively in the annotated reference material.
