# Supplementary material for: Towards a global understanding of the drivers of marine and terrestrial biodiversity
Source: PLoS One. 2020 Feb 5;15(2):e0228065. doi: 10.1371/journal.pone.0228065 (PMC7001915; doi:10.1371/journal.pone.0228065)
Supplement: S6 Fig — These are ternary plots that show binned medians of species richness at varying row-scaled relative ratios of the labeled drivers to one another. These plots highlight the converged or divergent nature of global species richness relative to drivers. The gray areas are where particular combinations of drivers are not observed in the dataset. Ann refers to interannual PP metrics and sub refers to intra-annual metrics. All temperature metrics are summaries of intra-annual data. These figures are offered as an alternative way to visualize Fig 3 in the main text. (DOCX) [file pone.0228065.s007.docx]

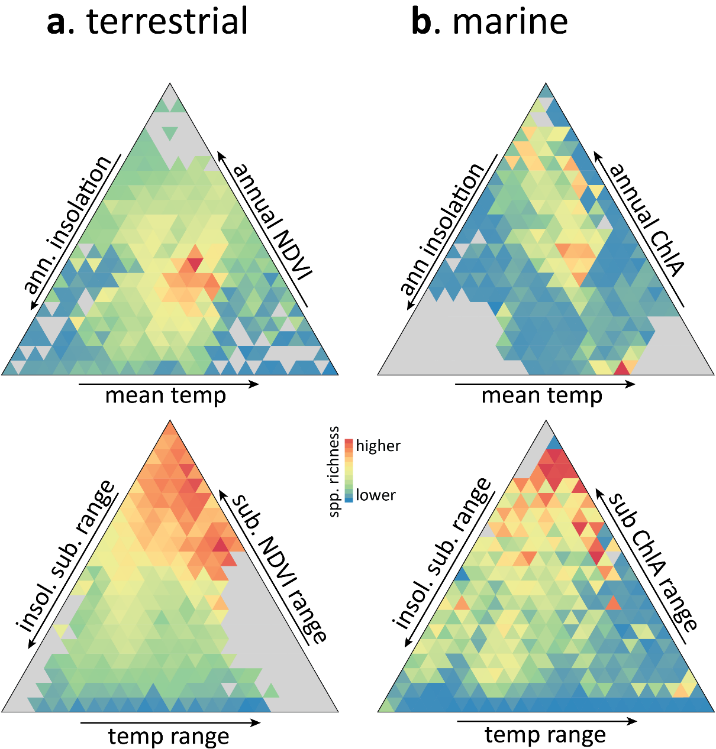


**Figure S6.** **Ternary plots offer exploration beyond pairwise comparison of drivers.** These are ternary plots that show binned medians of species richness at varying row-scaled relative ratios of the labeled drivers to one another. These plots highlight the converged or divergent nature of global species richness relative to drivers. The gray areas are where particular combinations of drivers are not observed in the dataset. Ann refers to interannual PP metrics and sub refers to intra-annual metrics. All temperature metrics are summaries of intra-annual data. These figures are offered as an alternative way to visualize Figure 3 in the main text.
