# Supplementary material for: Towards a global understanding of the drivers of marine and terrestrial biodiversity
Source: PLoS One. 2020 Feb 5;15(2):e0228065. doi: 10.1371/journal.pone.0228065 (PMC7001915; doi:10.1371/journal.pone.0228065)
Supplement: S7 Fig — The top row are terrestrial residuals, and the bottom are marine. On the left are the residuals of the full dataset with the color ramp utilized in the Fig 1C residual map. On the right are the residuals on the test set. (DOCX) [file pone.0228065.s008.docx]

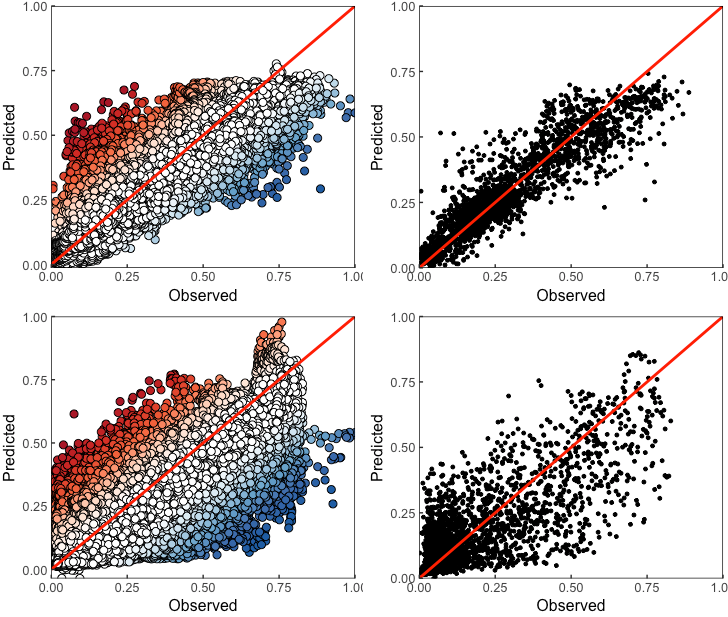


**Figure S7. Observed-versus-predicted for training and testing residuals.** The top row are terrestrial residuals, and the bottom are marine. On the left are the residuals of the full dataset with the color ramp utilized in the Figure 1c residual map. On the right are the residuals on the test set.
