# Supplementary material for: Towards a global understanding of the drivers of marine and terrestrial biodiversity
Source: PLoS One. 2020 Feb 5;15(2):e0228065. doi: 10.1371/journal.pone.0228065 (PMC7001915; doi:10.1371/journal.pone.0228065)
Supplement: S8 Fig — This is an alternative visualization of Fig 3. Each x and y axis now represents the two predictors (x is always elevation/depth). To this plot we added a marginal rug of observed values on both axes to show the distribution of raw data across the full domain of observations. (DOCX) [file pone.0228065.s009.docx]

**
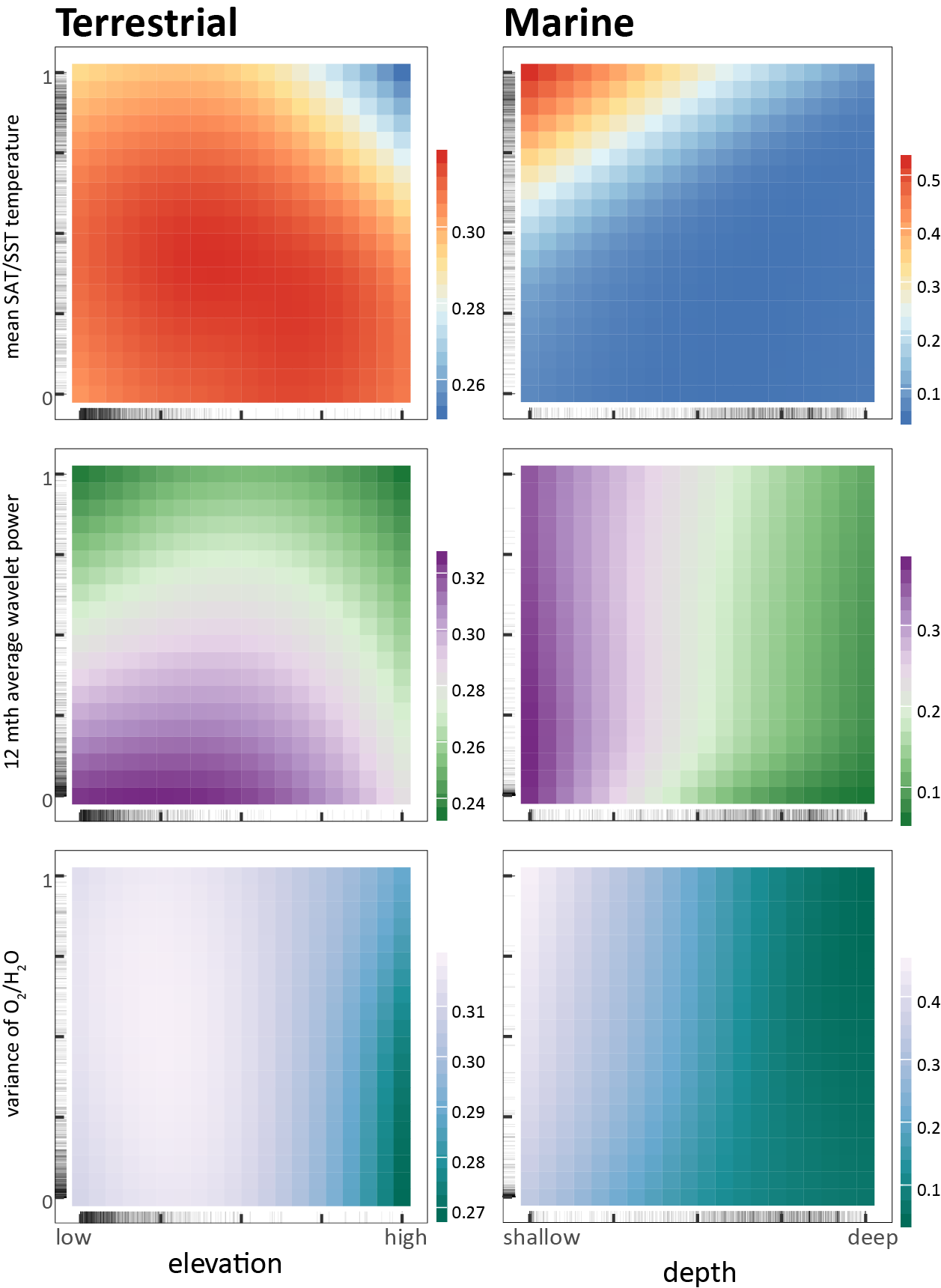
**

**Figure S8. 2D surface representations of the partial dependency plots from the main text.** This is an alternative visualization of Fig 3. Each x and y axis now represents the two predictors (x is always elevation/depth). To this plot we added a marginal rug of observed values on both axes to show the distribution of raw data across the full domain of observations.
