# Supplementary material for: Towards a global understanding of the drivers of marine and terrestrial biodiversity
Source: PLoS One. 2020 Feb 5;15(2):e0228065. doi: 10.1371/journal.pone.0228065 (PMC7001915; doi:10.1371/journal.pone.0228065)
Supplement: S9 Fig — Top row is observed, bottom is predicted. Left column is terrestrial, right is marine. All points are single points of richness from the observed values and modeled predictions. These are the raw points by which the Fig 1D estimates median richness in latitudinal bins. The color ramp is the same used in Fig 1A and 1B. (DOCX) [file pone.0228065.s010.docx]

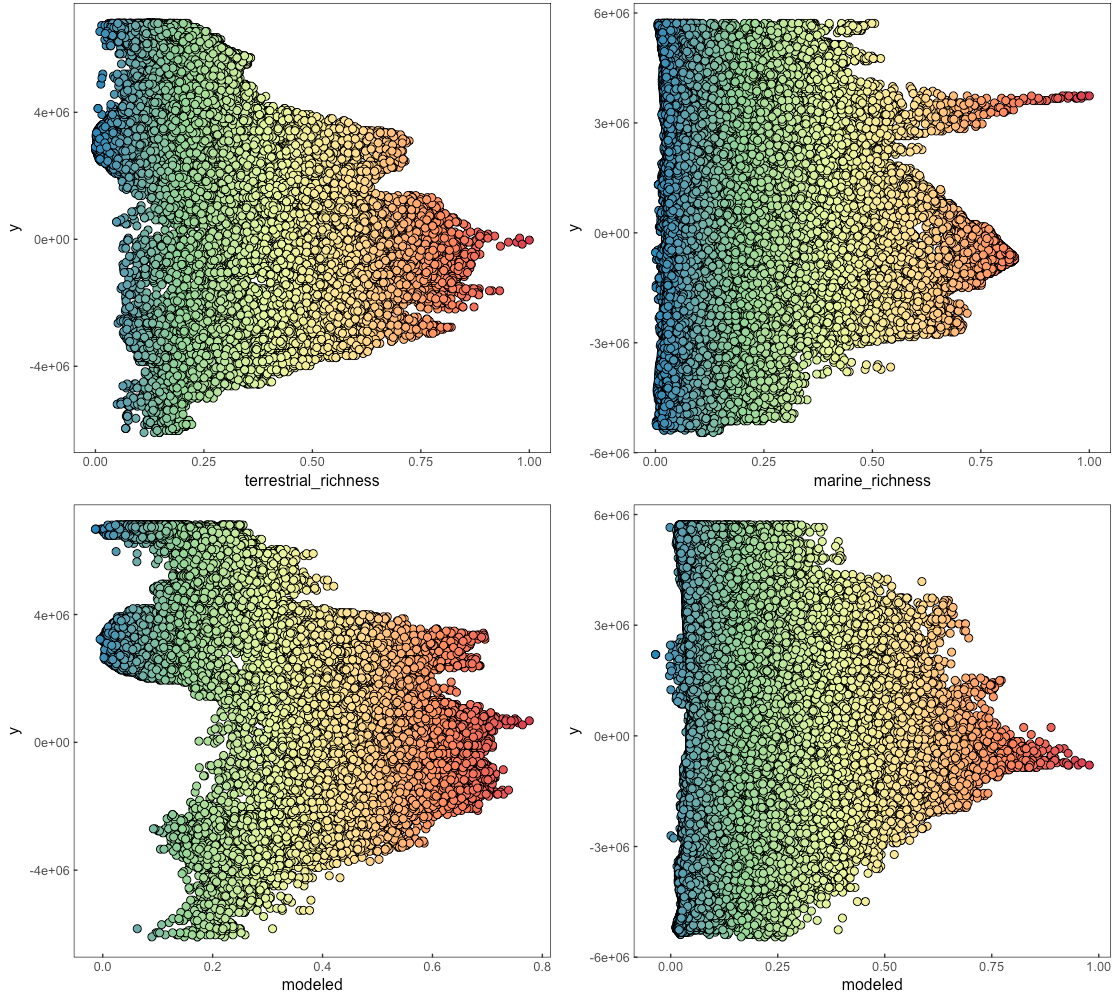


**Figure S9. Alternative visualization of latitudinal gradient.** Top row is observed, bottom is predicted. Left column is terrestrial, right is marine. All points are single points of richness from the observed values and modeled predictions. These are the raw points by which the Fig 1d estimates median richness in latitudinal bins. The color ramp is the same used in Fig 1a-b.
