# Supplementary material for: Towards a global understanding of the drivers of marine and terrestrial biodiversity
Source: PLoS One. 2020 Feb 5;15(2):e0228065. doi: 10.1371/journal.pone.0228065 (PMC7001915; doi:10.1371/journal.pone.0228065)
Supplement: S10 Fig — Species richness where the marine data does not contain invertebrate taxa. It has been suggested that varied patterns between domains may emerge if marine richness data does not contain invertebrate data in the same way the terrestrial data does not currently. These are the outputs of the analysis presented in the manuscript but conducted on marine species richness data without invertebrate taxa. The broad scale trends presented in the manuscript appeared to show little difference to those observed here. (DOCX) [file pone.0228065.s011.docx]

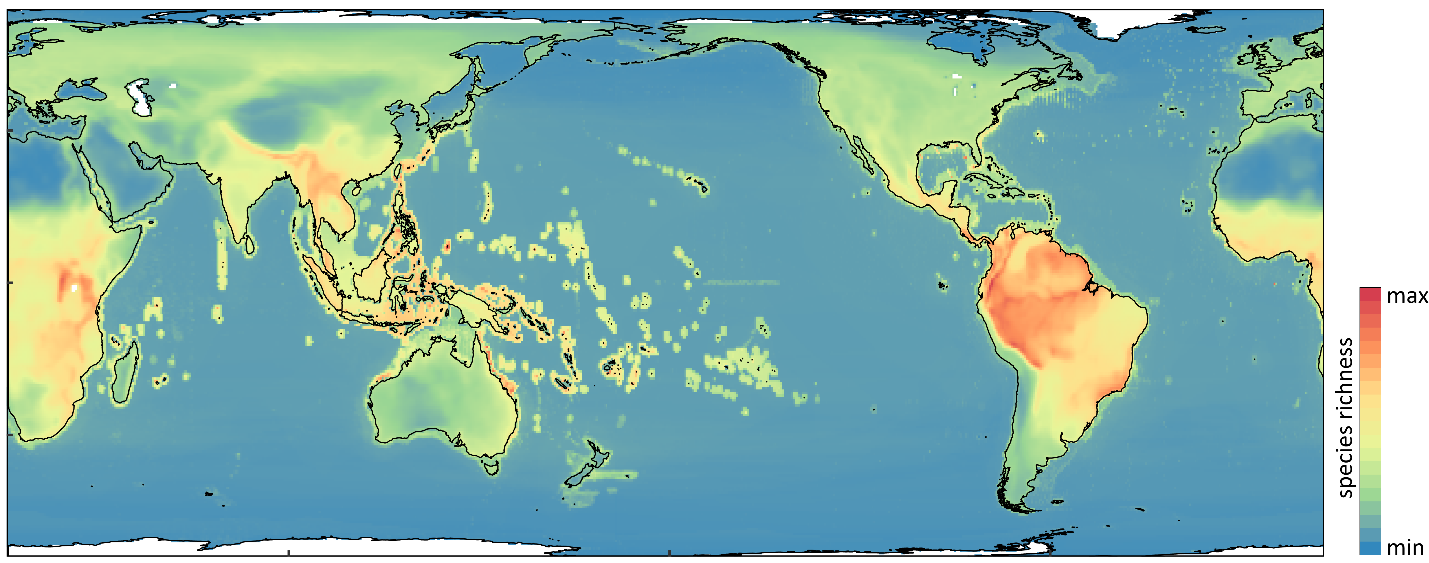


**Figure S10. Observed global species richness without invertebrates.** Species richness where the marine data does not contain invertebrate taxa. It has been suggested that varied patterns between domains may emerge if marine richness data does not contain invertebrate data in the same way the terrestrial data does not currently. These are the outputs of the analysis presented in the manuscript but conducted on marine species richness data without invertebrate taxa. The broad scale trends presented in the manuscript appeared to show little difference to those observed here.
