# Supplementary material for: Towards a global understanding of the drivers of marine and terrestrial biodiversity
Source: PLoS One. 2020 Feb 5;15(2):e0228065. doi: 10.1371/journal.pone.0228065 (PMC7001915; doi:10.1371/journal.pone.0228065)
Supplement: S12 Fig — These plots show the neural networks’ approximation of driver richness relationships when two inputs co-vary–a key product and advantage of the ANN modelling framework. In this example, we see how species richness is expected to respond across both different temperatures and seasonality in primary productivity in response to changing depth and elevation. See main text for comparison to marine richness data with full suite of available taxa. (DOCX) [file pone.0228065.s013.docx]

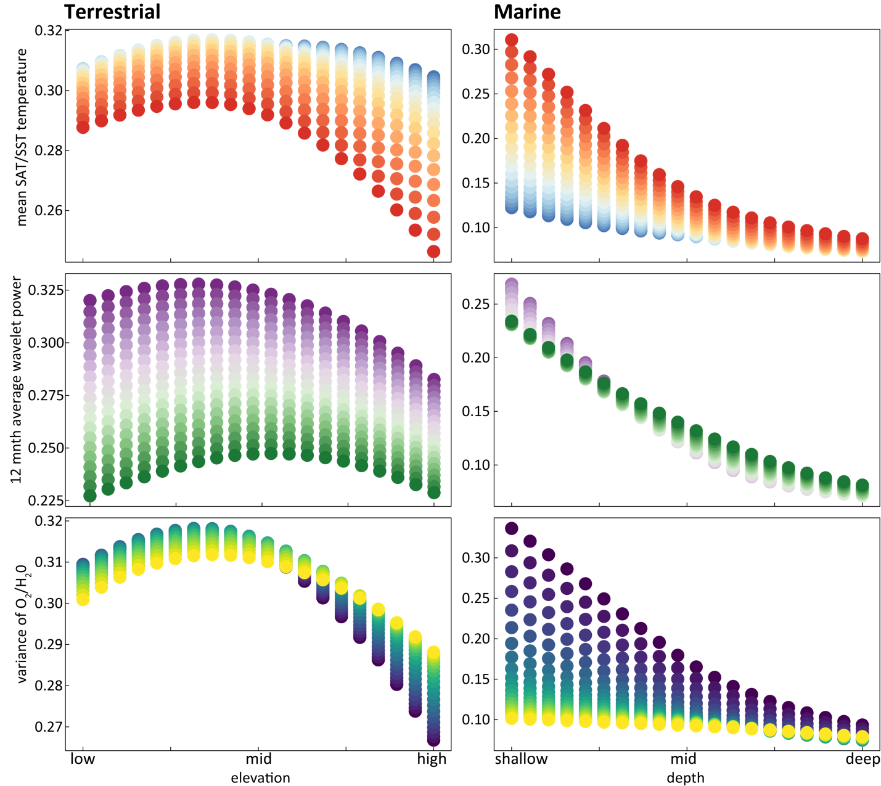


**Figure S12. Partial dependency plots visualizing model input interactions with species richness, when marine invertebrates are not included in the marine ANN model.** These plots show the neural networks’ approximation of driver richness relationships when two inputs co-vary – a key product and advantage of the ANN modelling framework. In this example, we see how species richness is expected to respond across both different temperatures and seasonality in primary productivity in response to changing depth and elevation. See main text for comparison to marine richness data with full suite of available taxa.
