# Supplementary material for: Towards a global understanding of the drivers of marine and terrestrial biodiversity
Source: PLoS One. 2020 Feb 5;15(2):e0228065. doi: 10.1371/journal.pone.0228065 (PMC7001915; doi:10.1371/journal.pone.0228065)
Supplement: S13 Fig — The hashed area represents the marine domain without invertebrate taxa. Here, (b) is identical to Fig 1D in the main text. The inclusion, or exclusion, of marine invertebrates, does not systematically alter the results of our analysis that compares modeled to observed richness in the marine ANN. As a note, neither of these issues affects the terrestrial analysis, which is separate, and for which no invertebrate data are available at the global scale. (DOCX) [file pone.0228065.s014.docx]

**
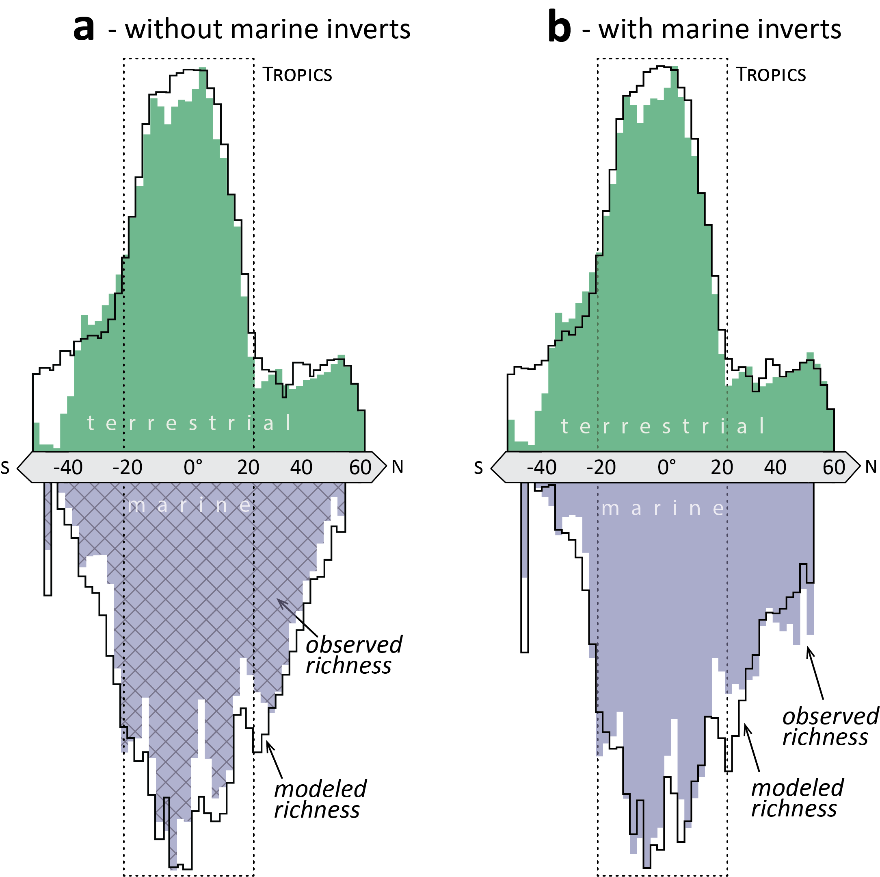
**

**Figure S13. Histograms of predicted versus observed richness across domains facetted by marine richness with or without invertebrates.** The hashed area represents the marine domain without invertebrate taxa. Here, (b) is identical to Figure 1d in the main text. The inclusion, or exclusion, of marine invertebrates, does not systematically alter the results of our analysis that compares modeled to observed richness in the marine ANN. As a note, neither of these issues affects the terrestrial analysis, which is separate, and for which no invertebrate data are available at the global scale.
