# Supplementary material for: Towards a global understanding of the drivers of marine and terrestrial biodiversity
Source: PLoS One. 2020 Feb 5;15(2):e0228065. doi: 10.1371/journal.pone.0228065 (PMC7001915; doi:10.1371/journal.pone.0228065)
Supplement: S14 Fig — The terrestrial domain remains the same as the main text analysis, whereas the marine domain is showing residuals of predicting marine richness without invertebrate taxa. The observed pattern in Fig 1C, where there is an overlap of species-poor marine regions and high velocity boundary currents is retained in this analysis. (DOCX) [file pone.0228065.s015.docx]

**
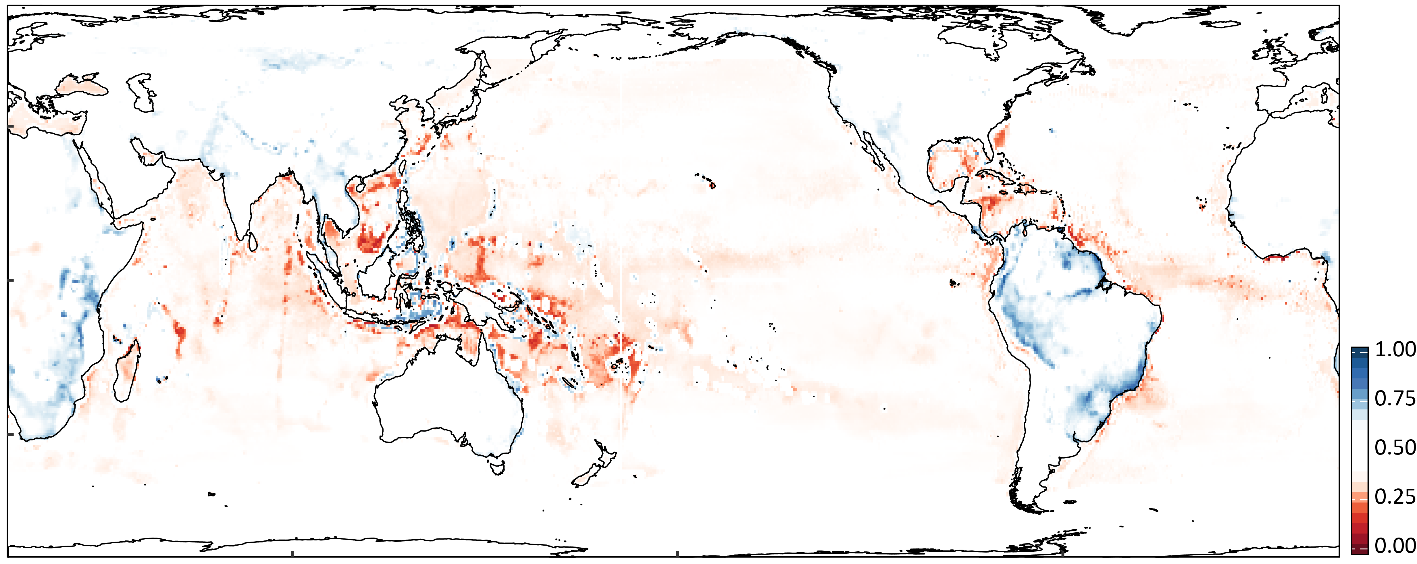
**

**Figure S14. Map of residuals from the model that did not include marine invertebrates.** The terrestrial domain remains the same as the main text analysis, whereas the marine domain is showing residuals of predicting marine richness without invertebrate taxa. The observed pattern in Figure 1c, where there is an overlap of species-poor marine regions and high velocity boundary currents is retained in this analysis.
