# Supplementary material for: Towards a global understanding of the drivers of marine and terrestrial biodiversity
Source: PLoS One. 2020 Feb 5;15(2):e0228065. doi: 10.1371/journal.pone.0228065 (PMC7001915; doi:10.1371/journal.pone.0228065)
Supplement: S15 Fig — The top four most important variables remain the same as the model of the species richness data containing invertebrate taxa. (DOCX) [file pone.0228065.s016.docx]

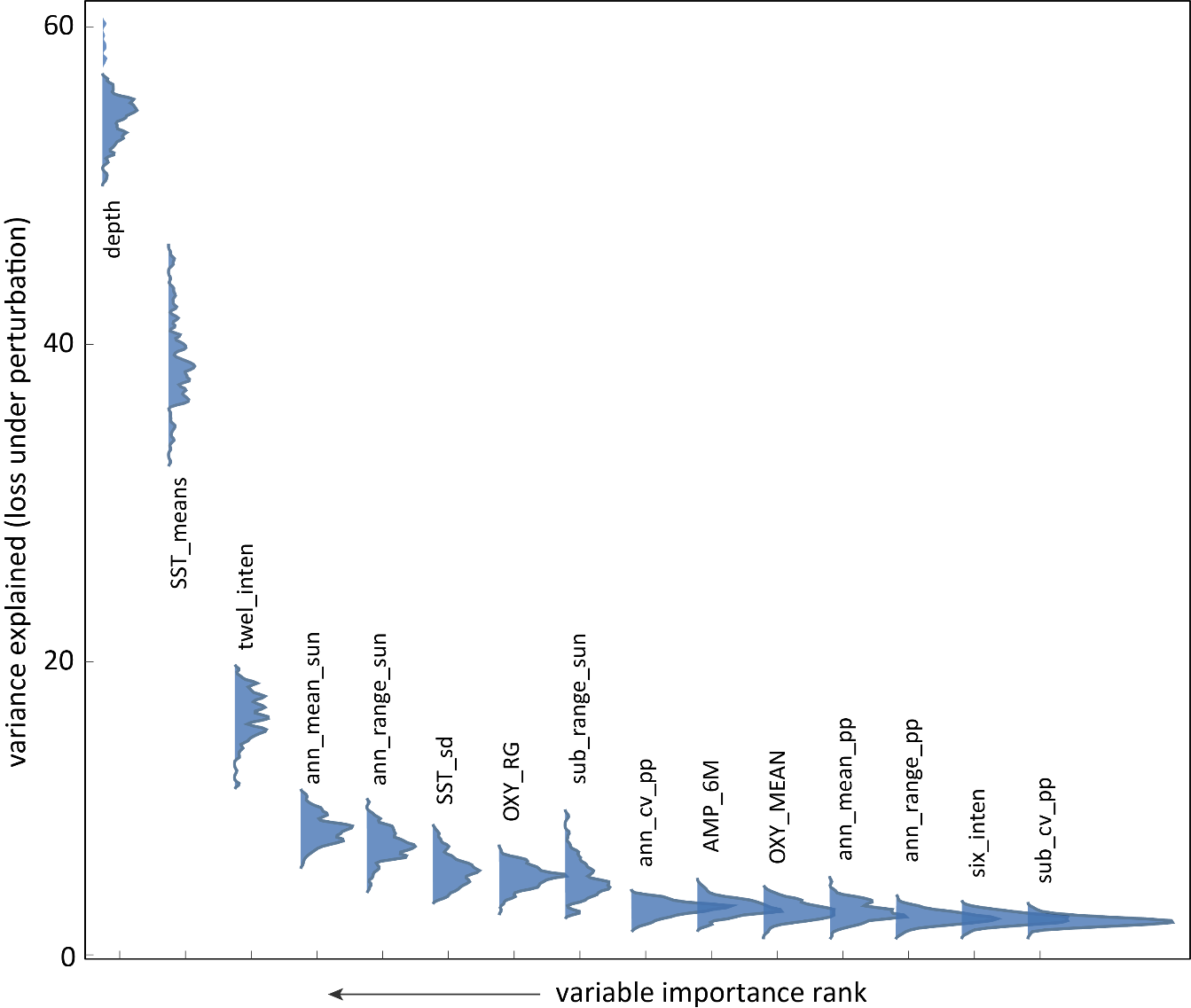


**Figure S15. Variable importance in marine domain without invertebrate taxa.** The top four most important variables remain the same as the model of the species richness data containing invertebrate taxa.
