# Supplementary material for: Towards a global understanding of the drivers of marine and terrestrial biodiversity
Source: PLoS One. 2020 Feb 5;15(2):e0228065. doi: 10.1371/journal.pone.0228065 (PMC7001915; doi:10.1371/journal.pone.0228065)
Supplement: S16 Fig — The above figure shows rarefaction or species accumulation curves for each 20° latitude bin for marine biodiversity when all species are included. Colors of 20° latitude bins correspond to rarefaction plot symbology below, where northern and southern hemispheres are split. This shows that sampling effort was not correlated with any spatial gradient of diversity. Thus, sampling did not significantly influence the observed biodiversity gradient depicted in Fig 1B and 1C. (DOCX) [file pone.0228065.s017.docx]

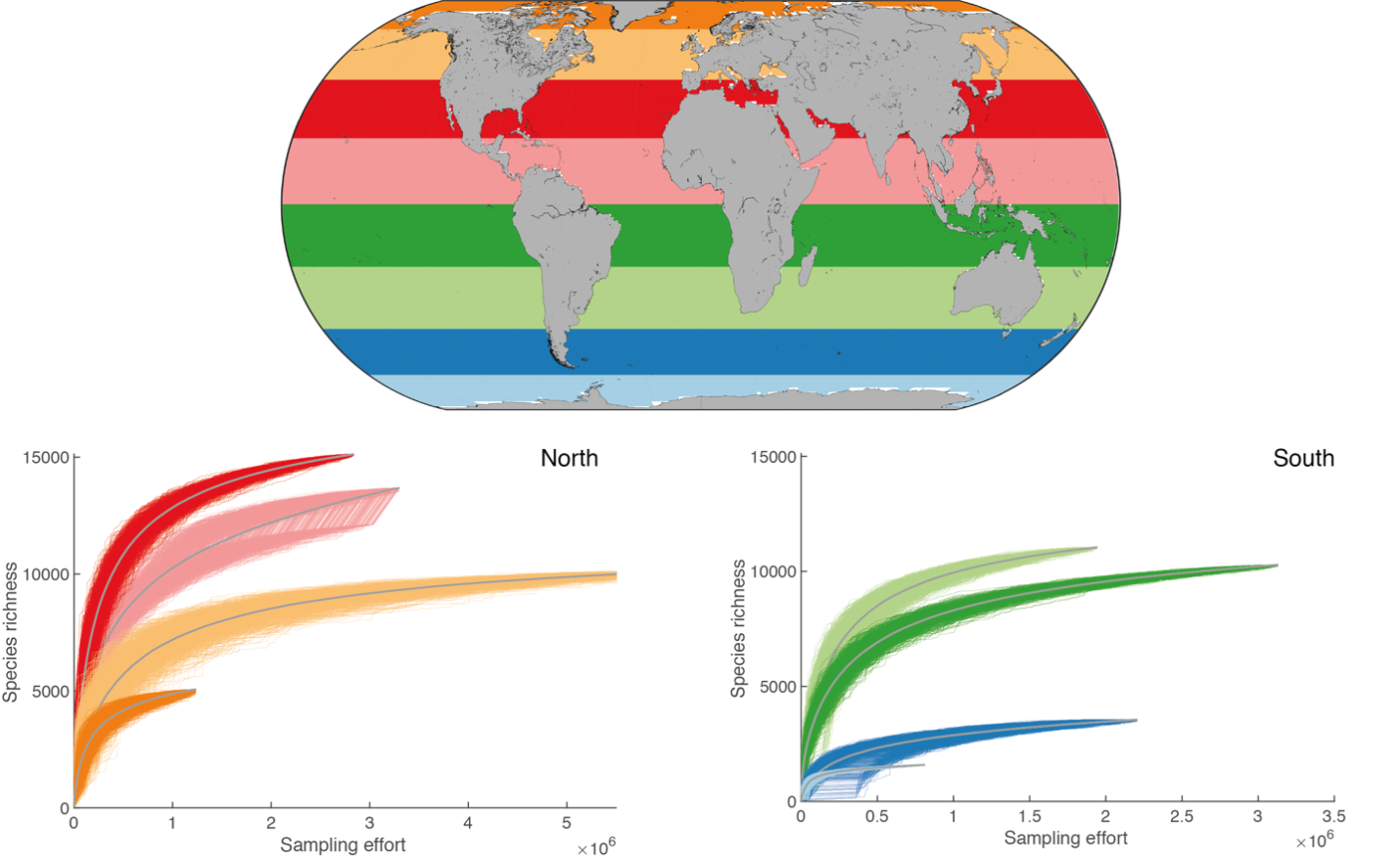


Figure S16. Assessing the potential impact of sampling bias on the marine species distributions. The above figure shows rarefaction or species accumulation curves for each 20° latitude bin for marine biodiversity when all species are included. Colors of 20° latitude bins correspond to rarefaction plot symbology below, where northern and southern hemispheres are split. This shows that sampling effort was not correlated with any spatial gradient of diversity. Thus, sampling did not significantly influence the observed biodiversity gradient depicted in Figure 1B-C.
