# Supplementary material for: Towards a global understanding of the drivers of marine and terrestrial biodiversity
Source: PLoS One. 2020 Feb 5;15(2):e0228065. doi: 10.1371/journal.pone.0228065 (PMC7001915; doi:10.1371/journal.pone.0228065)
Supplement: Attachment — Submitted filename: PLoS editorial comment_Biodiversity drivers.docx [file pone.0228065.s018.docx]

**PLoS editorial comment_Biodiversity drivers**

Overall, a really interesting and well written manuscript. My reading of this is that this is describing a ‘new’ method. If this is the case please be aware that PLoS has specific criteria for such papers as noted in the online submission guidelines (see below).

*PLOS ONE* will consider submissions that present new methods, software, databases, or tools as the primary focus of the manuscript if they meet the following criteria:

**Utility** 
The tool must be of use to the community and must present a proven advantage over existing alternatives, where applicable. Recapitulation of existing methods, software, or databases is not useful and will not be considered for publication. Combining data and/or functionalities from other sources may be acceptable, but simpler instances (i.e. presenting a subset of an already existing database) may not be considered. For software, databases, and online tools, the long-term utility should also be discussed, as relevant. This discussion may include maintenance, the potential for future growth, and the stability of the hosting, as applicable. 

**Validation** 
Submissions presenting methods, software, databases, or tools must demonstrate that the new tool achieves its intended purpose. If similar options already exist, the submitted manuscript must demonstrate that the new tool is an improvement over existing options in some way. This requirement may be met by including a proof-of-principle experiment or analysis; if this is not possible, a discussion of the possible applications and some preliminary analysis may be sufficient. 

**Availability** 
If the manuscript’s primary purpose is the description of new software or a new software package, this software must be open source, deposited in an appropriate archive, and conform to the [Open Source Definition](http://www.opensource.org/docs/osd). If the manuscript mainly describes a database, this database must be open-access and hosted somewhere publicly accessible, and any software used to generate a database should also be open source. If relevant, databases should be open for appropriate deposition of additional data. Dependency on commercial software such as Mathematica and MATLAB does not preclude a paper from consideration, although complete open source solutions are preferred. In these cases, authors should provide a direct link to the deposited software or the database hosting site from within the paper. If the primary focus of a manuscript is the presentation of a new tool, such as a newly developed or modified questionnaire or scale, it should be openly available under a license no more restrictive than CC BY.

PLoS offsets reference numbers with square brackets, not parentheses so please correct throughout the manuscript. The supporting information is also not referenced correctly or titled correctly; it should be S1 table etc, not the other way round. The results often drift into the present tense when the past tense should be used.

Line 34: Replace the en dash with a comma and write as ‘and have not’

Line 51: Write as ‘and is no longer’

Line 59: PLoS has supporting information, not supplemental material and this should be more specific to help the reader so please give something like S1 Table or S2 Figure as appropriate.

Line 66: The abbreviation ANN has not yet been established so please spell this out here along with the abbreviation

Lines 108-118: This paragraph is largely in the present tense but should be in the past tense

Line 156: Should be ‘were gathered’; also need to spell out IUCN and establish the abbreviation

Line 158: ‘were drawn’

Line 163: Can just write IUCN here as the abbreviation is established above

Line 168: I think this shold be ‘was selected and the thermal’

Line 175: Should be ‘were projected’

Line 176: what online repository? You should be specific where this can be found and move the link from the end of the paper to here

Line 180: PLoS has supporting information, not supplemental material and this should be more specific to help the reader so please give something like S1 Table or S2 Figure as appropriate.

Line 182: Again, be specific about where this can be found.

Line 186: Use NDVI here

Line 191: what is NPP? Need to spell this out.

Line 193: Should be ‘were taken’

Line 196: Ocean depth is the…’ this is very awkward as written. I think this should be similar to the above sentence “ocean depth data were taken’. Please make similar changes for the rest of the methods.

Line 201: Delete ‘ECMWF’ as this abbreviation is not used again in the manuscript

Line 201: Need to spell out SST and SAT

Line 204: Should be ‘data were’ and as noted above this is awkward as written so please rephrase

Line 205: delete ‘GPCC’ as this is not used again in the manuscript

Line 208: delete ‘CERES’ as this is not used again in the manuscript

Line 215: Need to establish the abbreviation ‘Chl-A’ where this is first mentioned

Line 216: Should be ‘S1 Table’

Line 224: ANN should be defined on line 66 so don’t need to do that again here

Line 232: Need a space between ‘scalable’ and the reference number

Line 238: Should be ‘were trained’

Line 267: Need a space between ‘inputs’ and the ref number

Line 273: Again, what code repository? Again, be specific

Line 289: Supporting information

Line 290: S1 Table

Line 291: S1-S9 Figs

Line 396: delete ANNs or just use the abbreviation, don’t use both
