# Supplementary material for: Towards a global understanding of the drivers of marine and terrestrial biodiversity
Source: PLoS One. 2020 Feb 5;15(2):e0228065. doi: 10.1371/journal.pone.0228065 (PMC7001915; doi:10.1371/journal.pone.0228065)
Supplement: Attachment — Submitted filename: Gagne_etal_Response_to_Reviewers.docx [file pone.0228065.s019.docx]

***RESPONSES TO REVIEWER COMMENTS***

***FOR GAGNE ET AL***

***“ON THE DRIVERS OF GLOBAL MARINE AND TERRESTRIAL BIODIVERSITY”***

***SUBMITTED TO PLoS ONE***

**EDITOR COMMENTS**

**E.1 I found this to be a really interesting paper that could be potentially far reaching… I am going to provide the authors with an opportunity to revise… it could be a really useful contribution…**

*Thank you for your comments and the opportunity to revise the manuscript at PLoS. We feel we have responded to the reviewers’ comments both in our revisions and in the comments below. Of note, we have (i) repositioned, prioritized, and bolstered the Methods section, and (ii) reframed what this study does and does not accomplish especially in the context of ANNs and the two domains. We hope this suffices, and again many thanks for making this manuscript better.*

**E.2 However… [it] does not meet the PLoS standard for methods papers… does not make it clear why this method is better… methods section really needs some substantial expansion**

*We have revised the paper and the supplement throughout for more details, clarity, and authoritative references (36 more added) to both explain and justify the methodological approach. The Methods section alone now is 2,200 words, has 18 citations, and is much more annotated and connected to the Supplemental material. Please also note that for analyses such as this the annotated scripts for computer code (in this case in R) are incredibly important and not emphasized enough. Even though not required, we provide this code, complete with annotated commentary in the paper’s online open-access repository (*<https://osf.io/jm7fn>*). For our approach, and for papers focused on methods, this code is critical for understanding the general framework as well as for understanding and reproducing the study. This is now overtly stated in lines 265-268.*

**REVIEWER 1 COMMENTS**

**1.1 This manuscript is an important contribution to the study of global patterns of biodiversity… I have been waiting to see such an application… Given the novelty… concerns need to be addressed.**

*Thank you for the encouraging words. We have now responded to all the reviewer concerns and now have a much-improved manuscript. This feedback is incredibly helpful.*

**1.2 The authors do demonstrate that a NN approach can improve understanding of driver interactions… [but] do not clearly… present why or how a [NN] can be better than using more traditional analyses…**

*Passages revised. We thank the reviewer for this feedback, and we have made attempts throughout to more overtly enumerate how ANNs compare and contrast to previous approaches. Please see in particular 75-95. Please also see responses to comments* ***2.9*** *and* ***2.10*** *below.*

**1.3 Despite my excitement to see the use of ANNs for this research question, I have concerns about the generality… As driver data are added or removed, does this change the results?**

*Thank you for the continued encouragement. Perhaps we misunderstood this comment, but as with all modeling approaches, the results will change as predictor variables are added. ANNs are no different than other models in this regard, they will only use the data they are fed. However, ANN will use a permutative approach to weight the contribution of each variable and will do this to find the best average or neural pathway. Therefore, compared to more traditional modeling approaches like GLMs or GAMMs, the ANN approach will have a more robust weighting scheme over all the environmental variables used to feed the model.*

**1.4 How sensitive are the results to differences in data processing (e.g., scaling)?**

*Passage revised. Scaling does not impact the model or its interpretation. Rescaling the data was simply done to facilitate model training by putting all model inputs on the same magnitude. We here selected the lowest scale gathered from the environmental results which allows us to keep the macro-ecological gradients. See lines 200-203. This is one noted area that ANNs tend to be a bit fussy as they benefit from being similarly scaled.*

**1.5 L255-260 the authors mention that ANNs can be used to parameterize interactions between variables without having to be explicitly defined, is this ever a draw back?**

*Passage revised and see response to* ***1.4*** *above. The main issue that surfaces here is the processing of the raw data inputs to the ANN. Both Random Forest models and NNs may require more extensive data pre-processing than more traditional methods where there are more formal parameterizations. The abilities of ANNs to detect and model interactions that are not explicitly defined is rarely a drawback. Spurious interactions can be parametrized in an ANN, but only in situations where a model is trained on a small dataset that is not a representative sample of a broader population. Which is not the case here. In the case of our dataset, and the chosen parameterization of the ANN, our approach is resilient to spurious interactions being trained in the model.*

**1.6 Species richness observations were scaled, but I did not see how the data were scaled…**

*Passage revised. Please see the responses to* ***1.4*** *and* ***1.5*** *above. All input (driver) and response (richness) variables were rescaled from 0-1. All the driver data were otherwise not transformed in any way. Only the richness data were log-transformed, please see line 142-143.*

**1.7 I am surprised by the lack of discussion of the latitudinal gradient… I recognize… authors are trying to make the point that analysis of direct drivers rather than proxies/surrogates is an advantage**

*Passage revised, please see lines 291-306 (to a lesser extent 115-136). Latitude is not itself a mechanism, but a proxy that summarizes the confluence of individual variables across space. Now that those variables are themselves more available at finer spatial and temporal scales and resolutions, it makes sense to model the mechanisms themselves directly. A primary advantage of this approach is that many of the underlying variables associated with latitude are shifting in amplitude rapidly. So, while the single variable of latitude may have helped historically in describing patterns of biodiversity, what latitude itself has meant for ecology and evolution is fundamentally altered. Therefore, the usefulness of latitude itself in forecasting the impacts of climate change are limited.*

**1.8 some of the implications or interpretations… would benefit from more explanation. Figure 1C leaves much to be discussed.**

*Passage revised. Please see lines 276-294 as well as the response to* ***1.9*** *and the associated revisions in the manuscript.*

**1.9 Are there driver datasets that the authors believe are missing?**

*As we state, we have made significant improvements to previous approaches by adding more variables and more descriptions of those variables. Please see revisions and additional references in lines 341-359. It’s a good point.*

**1.10 L34 I find ‘geographic domains’ unclear, is this meant to terrestrial/marine**

*Passage revised, see line 45. Yes, it is terrestrial/marine. In our revisions, we have been more careful in how we explain and use ‘domain’ and have examined this throughout.*

**1.11 L37 I think this sentence can be unpacked. The functional form of what? This is not immediately clear unless you have read the manuscript.**

*Passage revised for clarity.*

**1.12 L59 Why not include marine invertebrates in the main text results? They do not appear to change the results substantially…**

*Passage revised. We have combined two of the largest datasets on biodiversity in the marine and terrestrial realm, recognizing that species frequencies differ between land and sea. These species-specific differences lead to comparable datasets on biodiversity, yet they vary in terms of overall taxonomic comparison. We have limited our comparisons to consumers for this reason and feel this provides a best-available approach towards understanding drivers of biodiversity on land and in sea. Ultimately, we provide both results, but in the main text wanted to keep the terrestrial and marine domains as close to comparable as is possible.*

**1.13 why seek to unify the marine and terrestrial patterns of biodiversity? … The comparison… is insightful, but the framing of a unification of domains is unnecessary.**

*We have significantly reframed this approach throughout (see Title, line 49-57, etc.) to reflect a combined, not integrated or unified, approach. This is so we can compare and contrast between domains, which to some extent, are distinct.*

**1.14 L63 it is worth including the primary producers in at least a separate analysis… Especially given that you discuss the potential importance of structurally complex biogenic substrates**

*We excluded primary producers because including them would have created a problem of circularity in the model, since we have primary production as a predictor variable. We have revised the text to clarify this.*

**1.15 What is the collinearity between primary productivity measures (NDVI and Chl-A) and diversity of the primary producers?**

*See previous comment.*

**1.16 L67 What new information is provided about the driver relationships? Can these not be considered using traditional methods of analysis?**

*Please see comments and lines 75-95 in the text. All the interaction relationships are new information, and not published previously (at least by with both marine and terrestrial domain combined) and something that an ANN can provide unique insights to, and something that a GAMM cannot.*

**1.17 L76 What is the input-output relationship?**

*Passage revised, please see line 212*.

**1.18 L86 Why do you think that depth did not capture the overpredicted regions in oceanic habitats too deep for major coral reefs? … worth discussing the limitations in the driver data.**

*Passage revised, please lines 290-291.*

**1.19 What datasets are only available for surface waters and how does this affect the model’s ability to predict diversity patterns? (e.g., statements about the effect of O2… particularly Fig 2c?)**

A priori*, all remote sensed marine data are only available for surface waters. We have revised passages accordingly (lines 166-173) to make sure this is a limitation of the approach (lines 357-359), and of currently available data at the global scale*

**1.20 L88 Why would high-velocity current systems be more species-poor than expected?**

*Passage revised.*

**1.21 L91 What are the favorable conditions for species richness, those drivers which you identified in the previous paragraph?**

*See response to* ***1.7*** *above.*

**1.22 L93 What data do you show this pattern of habitable depths and temperatures? You jump to metabolic constraints without explaining this.**

*This section has been extensively rewritten.*

**1.23 This statement about patterns in spatial patterns of global fisheries production makes it seem like depth might be a proxy for a suite of other drivers. I am also unsure of the purpose of this comparison.**

*We have removed this sentence.*

**1.24 L133** **Synthesizing data across what kind of domain?**

*See above response to comment* ***1.10****. We have now defined domain more overtly.*

**1.25 L148 In what ways do the underlying datasets ultimately differ?**

*Passage revised.* *This is explained in lines 146-147.*

**1.26 L151 What was the resolution of these datasets?**

*Passages revised for clarity. All datasets are at 50×50 km.*

**1.27 L177 Provide reference to supplementary information**

*We have added* ***37*** *references to the main text in our revision, and as per journal instructions, there are no additional citations in the supplement that are not from the main text. So, the main text references serve for the supplement as well.*

**11.28 L229 What are perturbation and resampling and how do these help us understand driver effects…**

*Passage revised with more details. See lines 212-218. Similar to more traditional modeling approaches, these are model randomizations that serve as sensitivity analyses on the model outputs such as variable importance.*

**1.29 L345: Capitalize ‘science’ – and a general check over references, this always gets me too.**

*Revised. We have gone through all the references for such edits. Thank you!*

**1.30 Figure 1c: labelling the +, - on the scale could be replaced with over/under prediction to make it easier for the reader.**

*We debated this internally and went back and forth. Both are accurate, and in the end for simplicity’s sake we retained the notation in the graphic but provided more explanation in the caption. So, we have both.*

**1.31 Fig2a Are the predictors intensity/concentration/level of these drivers or are they variance?**

*Annual means -* *passage revised for clarity. See lines 543-544.*

**1.32 …presumably it is the original values rescaled from 0 to 1, but need to confirm and revise description to clarify…**

*Passage revised for clarity, but you are* *correct, the raw variable values are rescaled from 0-1.*

**1.33 Figure 3c: Where do you discuss this in the text?**

*Passage revised, please see lines 325-340.*

**1.34 It also does not make sense to look at the model effect of O2 variance in the deep ocean if the data for these depths were not used…**

*Please see response to* ***1.19*** *above.*

**1.35 Supplementary info: It would be helpful to reference specific figures in the supplement.**

*Agreed, and we have annotated the Methods and Results of the main text throughout.*

**1.36 Table S1… helpful to have the spatial and temporal resolution of the datasets included...**

*Fig. S1 maps the spatial resolution of all variables and Figs. S2-S3 display the statistical domains of all variables. We pulled from the full temporal extent available for all RS data of such resolution. This is described in the main text under each data section.*

**1.37 Figure S10: Is this the expected richness from the model?**

*Passage revised for clarity.*

**REVIEWER 2 COMMENTS**

**2.1 Gagne et al. present an exciting prospect of training emerging AI/ML techniques into global biodiversity assessment…**

*Thank you!*

**2.2 The authors have not properly addressed some of the major issues in using sparse point observation data for community level analyses.**

*Passage revised for clarity and further explanation. We developed a standardised database drawing from > 10 authoritative repositories of global marine species occurrences (OBIS, IOC, GBIF, Fishbase, IUCN, etc.). We then performed several rigorous quality control routines described in the Methods.*

**2.3 The authors have revisited analyses that have been undertaken… in the terrestrial space using a relatively underused method. They have not shown any value-add to using these new methods.**

*The aim of PLoS is rigor, not novelty. However, our analysis is novel in several respects. Here we are using the best-available distribution data for terrestrial data, which truthfully have been previously presented. However, we add new environmental drivers, new* metrics *of those drivers (that account for seasonality not just mean) and present a new modeling framework for their analysis. In addition to all of this, we combine this terrestrial analysis to a more novel marine analysis that itself contains an improved characterization of marine species occurrences. So, we are innovating in both domains, and we are doing that in the same study. This has not been done before either singly or in a combined form.*

**2.4 The authors have done an impressive job of… matching and filtering these data… However, I see nothing… to ensure… true species richness rather than… sampling bias.**

*Please see response to comment* ***2.2*** *above. Also, please see added Figure S13 that shows how we performed several analyses to evaluate the effect of sampling across latitudes. This represents both correlations between species richness and sampling effort and shows plots of accumulation curves binned in every 20° latitude. This shows that sampling effort was not correlated with any gradient of all diversity, either by latitude or taxonomic group (see table below). The accumulation curves of the filtered database further reveal that all biomes and latitudes were well sampled. Thus, sampling did not significantly influence the observed biodiversity gradient depicted in Fig. 1C. We nonetheless recognize that there is variation in sampling effort between ocean basins, with the Southern Atlantic and South Pacific Ocean being comparatively less sampled than the equatorial Pacific and the Northern Atlantic. Below is a table from a paper we currently have in review elsewhere (Reygondeau et al in revision) and is now cited in the main text:*

**2.5 They state that their database covers ~17% of marine species suggesting significant gaps in their aggregated richness data.**

*Passage revised. See lines 114-140. Costello et al 2017 (cited study) estimates that there are 245,000 named marine species listed in the WoRMs database. Our dataset here of 45,000 mapped species is the most ever published, and is up to date, and the most we currently have. Most studies cover far less, focusing on a single taxonomic group. Despite what we have here, clearly, we have more work to do as we only have mapped 18% of the known marine species.*

**2.6 If point observation data are appropriate for the marine models, are they are not used in the terrestrial models? GBIF contains 100s of thousands of terrestrial species that are not birds, mammals and amphibians (which only represent a tiny fraction of terrestrial diversity). The authors should be using the same kinds of primary data if they are to make inference between the two.**

*Passage revised. Data collection used in this article aim at representing the best quality and knowledge for both domains. Since the marine domain does not have standardized biodiversity dataset (with the exception of 2,000 fish species and 100 marine mammals), we here use point data after a quality control done for each species included in this study. However, standardized and controlled terrestrial datasets have been available for years. We did not include point data for the terrestrial to avoid introducing any bias in the analysis. We here aim to compare the drivers of distribution in both realms using up to date and controlled databases and not to collect all data and perform exploratory analysis. It is therefore a compromise between availability and quality – to an extent like all research* ☺

**2.7 The authors argue… advances in data streams and models will bear dividends in predicting the present and future distribution of biodiversity… Beyond the use of NNs… I don’t see many advances…**

*Extensive revisions made. While we do not see a specific comment here we can respond to… Briefly, the paper lays out a variety of novel results in the text, figures, and supplemental material that demonstrate areas of the globe that perform well/poor, presents new analyses of different metrics of environmental drivers (not just mean or max but seasonality), ranks drivers relative to their performance and shows the important multivariate interactions. This is of course all done in a modeling approach that has used more input and response data than done previously and is uniquely more scalable to future studies with even more data.*

**2.8 Very coarse… data when compared with modern global biodiversity assessments… more impressive to see this analysis carried out a spatial resolution more appropriate to the processes…**

*We do not know exactly what the reviewer is referring to by ‘assessment’, perhaps a single-species population assessment done within a confined geographic region? If so, perhaps a 50×50 km resolution wouldn’t be appropriate for, say, a warbler endemic to northern Michigan or a ROMS for a New England fishery. However, the aim of this paper is the collective influence of environmental drivers in predicting species richness at the global scale, and not a regional population assessment. So, the comparative criticism does not seen apt.*

**2.9 The paper needs to establish more clearly, where the actual benefits of ANN methods lie over a more common approach… Why is inference from ANN more appropriate?**

*Agreed, this could have been explained more clearly. Please see the added text and references in lines 76-96. Here we describe both the added features of ANNs and compare contrast to GAMMs and GLM. We also added 6 additional references to buttress those claims.*

**2.10 One of the big advantages of AI… integrate across VERY large datasets to draw inference… they make no mention of how many 50km pixels were used in the analysis**

*This is correct of course! Please see response to* ***2.9*** *above. We hopefully went the extra mile here too to more transparently enumerate the elements involved.*

**2.11 The methods need to be significantly expanded to improve clarity. In particular, the structuring of the model and the assumptions being made about the inputs…**

*We have made extensive revisions to the Methods enumerated throughout these comments.*

**2.12 Your data rely on IUCN range maps… do not even cover all vertebrates… a small fraction of biological diversity as a whole**

*Revisions made for clarity. We use the largest dataset ever compiled, and the best available data for terrestrial species, recognizing that the list is still not complete and will accrue species in future years. These data are rigorously vetted and expert reviewed. As stated in the text, for the marine domain, the occurrence data originated from several public sources of point observations - OBIS (*[*www.iobis.org*](http://www.iobis.org)*)*[*,*](http://www.iobis.org) *Intergovernmental Oceanographic Commission of UNESCO (ioc-unesco.org/), GBIF (*[*www.gbif.org*](http://www.gbif.org)*), Fishbase (*[*www.fishbase.org*](http://www.fishbase.org)*), the Coastal and Oceanic Plankton Ecology Production and Observation database (*[*www.st.nmfs.noaa.gov/plankton*](http://www.st.nmfs.noaa.gov/plankton)*), the Jellyfish Database Initiative (*[*http://condonlab.weebly.com/*](http://condonlab.weebly.com/)*), as well as the IUCN (*[*http://www.iucnredlist.org/technical-documents/spatial-data*](http://www.iucnredlist.org/technical-documents/spatial-data)*).*

**2.13 L52-3 Rephrase.**

*Done! Agreed that it needed some smoothing out.*

**2.14 L66 Define ANN**

*Changes made.*

**2.15 L98-100 While this may be true. It’s also true that these are the areas most easily studied, and your results represent a potential sampling bias in the underlying datasets**

*Please see response to comment* ***2.4*** *and new Figure S13 on sampling bias*

**2.16 Fig S13 Your terrestrial results with/without marine invertebrates… modelled within the same network… explain more clearly why this is appropriate…**

*Perhaps the reviewer is misunderstanding that we do not have invertebrate data in the terrestrial realm, simply because those data aren’t available at the global scale. Furthermore, the ANNs for each realm are completely distinct, so they are not modelled in the same network. We tried to make this clearer in the text - thanks.*
