# Supplementary material for: Towards a global understanding of the drivers of marine and terrestrial biodiversity
Source: PLoS One. 2020 Feb 5;15(2):e0228065. doi: 10.1371/journal.pone.0228065 (PMC7001915; doi:10.1371/journal.pone.0228065)
Supplement: Attachment — Submitted filename: PLoS editorial comment_Biodiversity drivers (R1).docx [file pone.0228065.s020.docx]

**PLoS editorial comment_Biodiversity drivers (R1)**

The authors refer to supporting information incorrectly. As noted in the online submission guidelines, supporting information should be referred to as ‘S1 Table’, ‘S2 Figure’ etc (not Figure S2). Please correct throughout manuscript.

The authors also introduce far too many abbreviations that are only used once again (e.g. GEBCO, ECMWF, GPCC, WOA and CERES) which gets very confusing. The PLoS rule is that they must be used at least three times, otherwise do not introduce them.

The Results also switch between the current and past tense and should all be in the past tense.

Line 64: PLoS does not have ‘supplemental information’ it has ‘supporting information’

Line 75: Should be ‘and in the sea’

Line 76: Do not capitalise ‘artificial neural networks’

Line 89: Spell out GLMs and GAMMs here (no abbreviations needed as they are not used at least 3 times again)

Line 91: Spell out GAMMs here

Line 110: Spell out IUCN

Line 117: Delete ‘(IUCN)’ and just spell it out.

Line 136: Should be ‘S16 Figure’

Line 148: Should be ‘S10-S15 Figures’

Line 149: Replace the dashes with commas

Lines 156-157: Should be ‘S1 Table and S1-S3 Figures’

Line 158: Introduce the abbreviation ‘Chl-A’ here

Lines 167-182: As noted above, these abbreviations should not introduced (e.g. GEBCO, ECMWF, GPCC, WOA and CERES)

Line 192: Should be ‘S1 Table’

Line 298: Something wrong here ‘but we do not detect’…..please correct

Line 299: Should be ‘where there are’

Line 309: Should be ‘climate change is’

Line 319: Should be ‘in response’

Line 324: S4 Figure and S6 and S8 Figures

Line 352: ‘pipelines’ is a bit strange here, is there another word that could be used?

Line 391: S1 Table, S1-S15 Figures
