# Supplementary material for: Towards a global understanding of the drivers of marine and terrestrial biodiversity
Source: PLoS One. 2020 Feb 5;15(2):e0228065. doi: 10.1371/journal.pone.0228065 (PMC7001915; doi:10.1371/journal.pone.0228065)
Supplement: Attachment — Submitted filename: PLoS editorial comment_Biodiversity drivers (R1).docx [file pone.0228065.s021.docx]

**PLoS editorial comment_Biodiversity drivers (R1)**

**The authors refer to supporting information incorrectly. As noted in the online submission guidelines, supporting information should be referred to as ‘S1 Table’, ‘S2 Figure’ etc (not Figure S2). Please correct throughout manuscript.**

*Done! Thanks.*

**The authors also introduce far too many abbreviations that are only used once again (e.g. GEBCO, ECMWF, GPCC, WOA and CERES) which gets very confusing. The PLoS rule is that they must be used at least three times, otherwise do not introduce them.**

*Changes made.*

**The Results also switch between the current and past tense and should all be in the past tense.**

*All changed to past tense. Thanks.*

**Line 64: PLoS does not have ‘supplemental information’ it has ‘supporting information’**

*Done!*

**Line 75: Should be ‘and in the sea’**

*revised*

**Line 76: Do not capitalise ‘artificial neural networks’**

*We debated this, and you are correct.*

**Line 89: Spell out GLMs and GAMMs here**

*Done.*

**Line 91: Spell out GAMMs here**

*Revised*

**Line 110: Spell out IUCN**

*Changes made*

**Line 117: Delete ‘(IUCN)’ and just spell it out.**

*Done!*

**Line 136: Should be ‘S16 Figure’**

*Done!*

**Line 148: Should be ‘S10-S15 Figures’**

*Done!*

**Line 149: Replace the dashes with commas**

*Done!*

**Lines 156-157: Should be ‘S1 Table and S1-S3 Figures’**

*Done!*

**Line 158: Introduce the abbreviation ‘Chl-A’ here**

*Good idea.*

**Lines 167-182: As noted above, these abbreviations should not introduced (e.g. GEBCO, ECMWF, GPCC, WOA and CERES)**

*All changes made.*

**Line 192: Should be ‘S1 Table’**

*Done!*

**Line 298: Something wrong here ‘but we do not detect’…..please correct**

*Deleted.*

**Line 299: Should be ‘where there are’**

*Correct, this has been changed.*

**Line 309: Should be ‘climate change is’**

*Done!*

**Line 319: Should be ‘in response’**

*Revised.*

**Line 324: S4 Figure and S6 and S8 Figures**

*Done!*

**Line 352: ‘pipelines’ is a bit strange here, is there another word that could be used?**

*Revised.*

**Line 391: S1 Table, S1-S15 Figures**

*Done!*
